# Intermittent preventive treatment of malaria delivered to primary schoolchildren provided effective individual protection in Jinja, Uganda: secondary outcomes of a cluster-randomized trial (START-IPT)

**DOI:** 10.1186/s12936-019-2954-0

**Published:** 2019-09-18

**Authors:** Andrea M. Rehman, Catherine Maiteki-Sebuguzi, Samuel Gonahasa, Jaffer Okiring, Simon P. Kigozi, Clare I. R. Chandler, Chris Drakeley, Grant Dorsey, Moses R. Kamya, Sarah G. Staedke

**Affiliations:** 1 Department of Infectious Disease Epidemiology, LSHTM, London, UK; 2 Infectious Diseases Research Collaboration, PO Box 7475, Kampala, Uganda; 3 Department of Global Health & Development, Department of Clinical Research, LSHTM, London, UK; 4 Department of Infection and Immunity, LSHTM, London, UK; 5 Department of Medicine, University of California, San Francisco, USA; 6 School of Medicine, Makerere University College of Health Sciences, Kampala, Uganda; 7 Department of Clinical Research, London School of Hygiene & Tropical Medicine (LSHTM), London, UK

**Keywords:** Malaria, Intermittent preventive treatment, Dihydroartemisinin–piperaquine, Schoolchildren, Cluster-randomised trial

## Abstract

**Background:**

Intermittent preventive treatment (IPT) of malaria is recommended as policy for certain high-risk populations, but not currently for schoolchildren. A cluster-randomized trial was conducted to evaluate the effect of IPT with dihydroartemisinin–piperaquine (DP) on primary schoolchildren in Jinja, Uganda. Results of the impact of IPT of schoolchildren on community-level transmission have been reported previously. Here, secondary outcomes from a school-based survey are presented.

**Methods:**

Eighty-four clusters (one primary school plus 100 households) were randomized to intervention and control (1:1 ratio). Participants from intervention schools received monthly IPT with DP for up to 6 rounds (June–December 2014). At endline (November–December 2014), randomly selected children from all 84 schools were surveyed (13 per school) and thick blood smears were done. Those with fever or history of fever were tested with rapid diagnostic tests (RDTs) for malaria. Haemoglobin was measured in every fifth participant. Outcome measures included prevalence of asexual parasites and gametocytes (by microscopy), and prevalence of anaemia. Prevalence outcomes were analysed using generalized linear Poisson models with log link function, incorporating a cluster-level random intercept and quantified using prevalence risk ratios.

**Results:**

Among 23,280 students listed on the 42 intervention school registers, 10,079 (43.3%) aged 5–20 years were enrolled into the IPT intervention and received at least one dose of DP; of these, 9286 (92.1%) received at least one full (3-day) course. In total, 1092 children were enrolled into the final school survey (546 per arm) and had a thick blood smear done; of these, 255 had haemoglobin measured (129 intervention, 126 control). Children in the intervention arm were less likely to have asexual parasites (9.2% intervention vs 44.1% control, adjusted risk ratio [aRR] 0.22 [95% CI 0.16–0.30] p < 0.001), gametocytes (3.1% intervention vs 9.5% control, aRR 0.34 [95% CI 0.20–0.56] p < 0.001), fever (20.2% intervention vs 56.2% control, aRR 0.35 [95% CI 0.25–0.50] p < 0.001), or symptomatic malaria (5.1% intervention vs 35.7% control, aRR 0.14 [95% CI 0.08–0.26] p < 0.001). Prevalence of anaemia and mean haemoglobin were similar in both study arms.

**Conclusions:**

School-aged children are a major reservoir of malaria parasites. Delivering IPT to schoolchildren would benefit individual children and may reduce transmission. School-based IPT could help to intensify malaria control toward elimination, and should be considered for policies and programmes.

*Trial registration* Clinicaltrials.gov (NCT02009215), Registered 11 December 2013. https://clinicaltrials.gov/ct2/show/NCT02009215

## Background

Malaria remains a major global health problem. Despite malaria control achievements over the last 20 years [1], recent data suggest that the global response and successful control of malaria may have plateaued, particularly in Africa [2]. According to the World Health Organization (WHO), the number of malaria cases reported from the ten highest burden African countries, including Uganda, increased by 3.5 million in 2017 [2]. In Uganda, efforts to scale-up coverage of malaria control interventions, through mass distribution of long-lasting insecticidal nets (LLINs), indoor residual spraying of insecticides (IRS), and effective case management with artemisinin-based combination therapy (ACT), have yielded positive results [3, 4]. However, malaria control gains have been difficult to sustain [5, 6] and the burden of malaria remains high [2, 7]. Innovative measures have been called for to achieve sustainable malaria control in Uganda and elsewhere in Africa [8, 9].

Children under five are at high risk of malaria in endemic areas and have typically been targeted for malaria control interventions, along with pregnant women. However, this approach overlooks school-aged children whose burden of disease is also important [10]; they are at risk for asymptomatic malaria infections [11], often have the highest parasite prevalence within populations [12, 13], and are important contributors to the infectious reservoir for onward transmission of malaria [14, 15]. Moreover, as malaria control interventions are scaled-up, and transmission intensity and consequently the level of acquired immunity in the population fall, the peak age of clinical malaria may shift from the very young, to include older school-aged children [16]. Thus, malaria morbidity and mortality may paradoxically rise in school-aged children as malaria is controlled, highlighting the need to focus on this age group as malaria control intensifies [17].

Intermittent preventive treatment (IPT) is a well-established malaria control intervention, which is recommended for pregnant women and infants. IPT of malaria in children under-five has also been operationalized as seasonal malaria chemoprevention (SMC) in West Africa. Chemoprevention is not currently policy for school-aged children, but extending SMC programmes to include older children has been investigated within the Sahel region of Africa [18, 19]. Evidence from Uganda and elsewhere suggests that IPT of malaria in schoolchildren provides significant health benefits and may improve cognitive function [20–23]. IPT of school-aged children also has the potential to reduce the infectious reservoir [22, 24]. Although momentum for chemoprevention of school-aged children is growing, more evidence is needed to help guide policy-makers.

To further evaluate IPT of malaria in schoolchildren, a cluster randomized trial was conducted in Jinja, Uganda in 2014–2015. Children enrolled from intervention schools received monthly IPT with dihydroartemisinin-piperaquine (DP) for up to 6 rounds of treatment. The primary objective of the trial was to evaluate the impact of IPT of schoolchildren on community-level indicators of malaria transmission, as measured in a post-intervention cross-sectional community survey and continuous entomological surveillance; these results have been reported previously [24]. Here, the secondary outcomes from school-based surveys are presented, adding to the evidence base for chemoprevention in older, school-aged children.

## Methods

### Study site

**Fig. 1:**
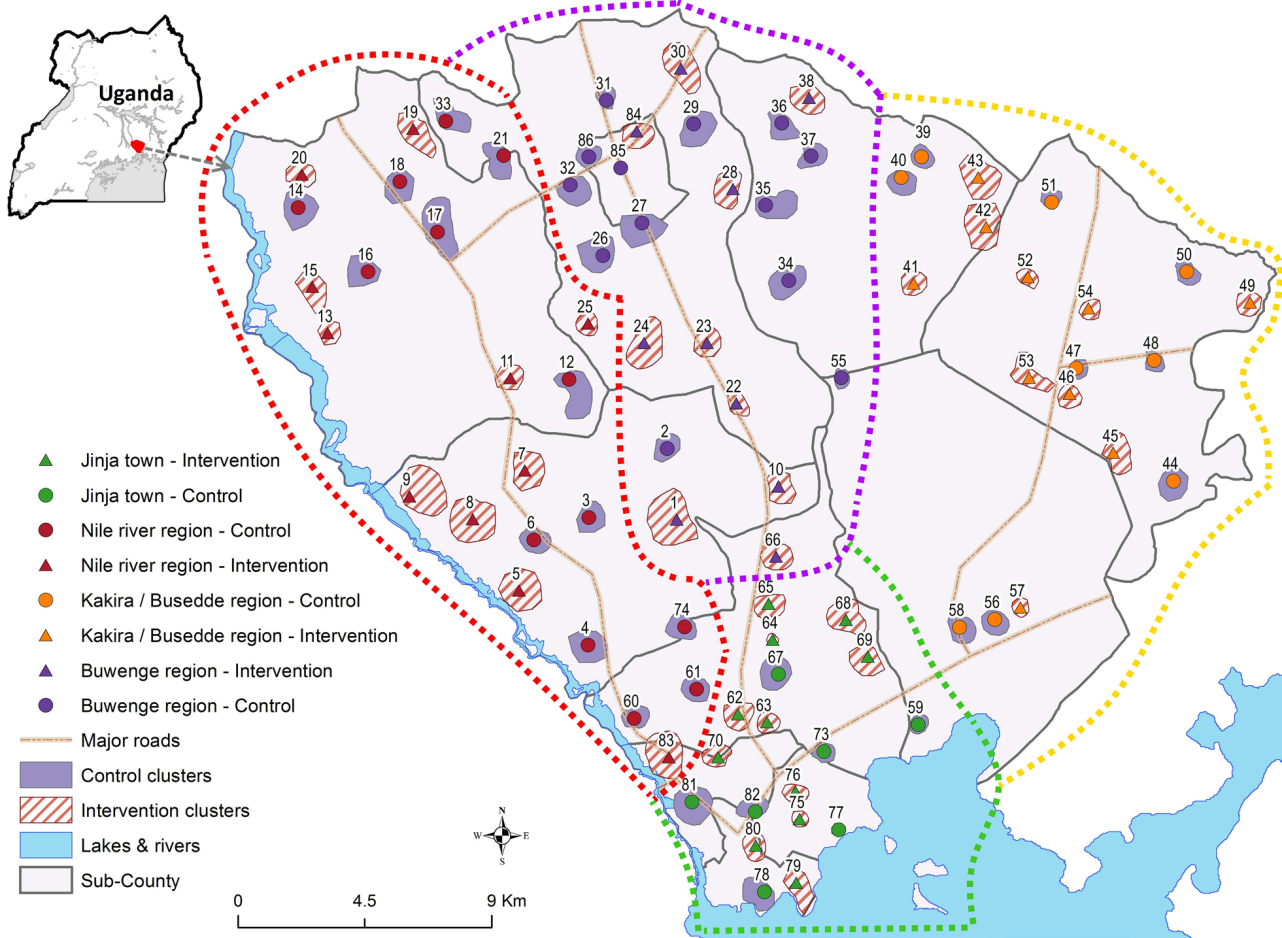
Map of the study area

The study was carried out in Jinja district in eastern Uganda, where malaria transmission is perennial (Fig. 1). At baseline, the annual entomologic inoculation rate (assessed from January to April 2014) was 58.9 infective bites per person per year [24], and parasite prevalence in children enrolled in intervention schools (assessed from April to June 2014) was 43.2% (95% confidence interval [CI] 36.9–49.7%) with substantial heterogeneity between schools (ranging from 0 to 75%).

### Clusters and randomization

Digitally enumerated maps were used to define clusters, including one primary school plus the 100 closest households surrounding the school [24]. One school was included per cluster and a buffer zone of 500 m was implemented between clusters. Public schools were prioritized. In total, 84 schools were included (72 public and 12 private schools). Clusters were assigned randomly in a 1:1 allocation ratio to intervention or control in a parallel design by the trial statistician based in London. Restricted randomization was applied using Stata/SEv12 software aiming to balance assignment of trial arms between sub-counties, and school type (public or private). Allocation of primary schools to the two study arms was not masked.

### IPT intervention

Prior to the study, team members met with key stakeholders in health and education at the national and district levels to sensitize them to the trial using an information sheet. Verbal consent to participate was obtained from headteachers of all schools, and copies of the school registers were obtained from intervention schools. Recruitment for the IPT intervention was carried out continuously from March to December 2014.

Study personnel met with parents/guardians to review initial eligibility criteria, including: (1) able to locate parent/guardian, (2) enrolled in intervention school, (3) age ≥ 5 years (4) no known allergy to DP, (5) no menarche (female students), (6) no history of cardiac problems or fainting, (7) no family history of long QT syndrome, (8) not currently using medications known to prolong the QT interval, and (9) willingness of parent/guardian to provide written informed consent. If these criteria were met, children were reviewed individually at school by study personnel for the final criteria, including: (1) able to locate student, (2) no menarche (female students), (3) weight ≥ 11 kg, and (4) provision of written assent by student (aged 8 years or above). Children who passed screening underwent a brief physical examination, including measurement of weight and were fingerprinted to facilitate identification for future IPT treatment.

Participants in the IPT intervention received DP (Duo-Cotexcin, Holley Cotec Pharmaceuticals) monthly for up to 6 rounds of treatment (June to December 2014). DP was administered by study personnel orally once daily for 3 days, using full-strength DP tablets (40/320 mg), according to weight-based guidelines (11–20 kg: 40 mg/320 mg daily; 21–30 kg: 60 mg/540 mg daily; 31–40 kg: 80 mg/640 mg daily; ≥ 41 kg: 120 mg/960 mg daily). All treatments were directly observed. The trial was open-label, and no placebo treatment was provided in the control schools.

### School survey

A cross-sectional survey of children from each participating school was conducted toward the end of the intervention (November–December 2014). A random sample of children was selected from each school register. Recruitment and screening were stratified by class, to ensure equitable distribution of participants from all classes (P1–P7), until the target sample size of children per cluster was reached. A similar survey was conducted at baseline prior to the intervention delivery and was used to inform sample size calculations.

Study personnel invited parents/guardians of selected children for a meeting at the school to screen for eligibility criteria, including: (1) enrolled in a participating primary school, and (2) agreement of parents/guardians to provide informed consent. If these criteria were met, study personnel interviewed each child individually at school for the final criteria, including: (1) able to locate student and (2) provision of written assent by student (aged 8 years or above). Children were excluded if they could not be located on more than 3 occasions.

If selection criteria were met, a brief questionnaire was administered to collect information on age, gender, bed net use, location of residence, and a brief food history. Participants had their temperature measured, and a finger-prick blood sample was obtained for a thick blood smear and haemoglobin measurement (in every fifth participant) using a portable spectrophotometer (HemoCue, Anglom, Sweden). RDTs were performed on participants with fever (tympanic membrane temperature of ≥ 38.0 °C) or history of fever in the past 48 h (*CareStart*™ Malaria HRP-2 *P. falciparum*; ACCESSBIO). Children with a positive RDT were treated with artemether–lumefantrine unless they exhibited signs of severe malaria, in which case they were referred to appropriate health facilities. Detailed records of school attendance were collected from both intervention and control schools.

### Laboratory procedures

Thick blood smears were stained with 2% Giemsa for 30 min and read by experienced laboratory technologists, who were not aware of study arm assignments. Parasite and gametocyte densities were calculated from thick blood smears by counting the number of asexual parasites and gametocytes, respectively, per 200 leukocytes (or per 500, if the count was less than 10 parasites or gametocytes per 200 leukocytes), assuming a leukocyte count of 8000/μl. A thick blood smear was considered negative when the examination of 100 high power fields did not reveal asexual parasites or gametocytes. For quality control, all slides were read by a second microscopist and a third reviewer settled discrepant readings.

### Outcome measures

The primary outcome among school children (a key secondary outcome of the trial) was prevalence of asexual parasitaemia, as measured by microscopy of individual blood smears. Secondary outcomes were prevalence of gametocytaemia, prevalence of anaemia [25], mean haemoglobin, and school attendance.

### Sample size estimates

Sample size was determined for the trial’s secondary outcome, smear positive microscopy among school children, and was refined in October 2014 after results from the baseline survey were available. Thirteen randomly selected children in each of the 84 clusters (1092 total), had over 80% power at significance level 5% to detect a relative reduction in parasite prevalence of 35%, corresponding to an absolute difference in parasite prevalence of 11.5% (33% vs 21.5%), assuming a coefficient of variation between clusters of 0.50.

### Statistical analysis

A secondary objective of the trial was to evaluate the impact of IPT for malaria in schoolchildren on clinical malaria indicators, aiming to test the hypothesis that the prevalence of asexual parasitaemia would be lower in the children enrolled in intervention schools than in those enrolled in control schools. The plans for the analysis presented here were outlined a priori in the statistical analysis plan for the trial. Data were analysed at the individual-level due to the large number of clusters per trial arm [26] and statistical methods which accounted for within-cluster correlation were used in all analyses. Prevalence outcomes were analysed using generalized linear Poisson models with log link function [27], incorporating a cluster-level random intercept and quantified using prevalence risk ratios. For other secondary quantitative outcomes, linear regression models were used. The effect of the intervention was quantified by calculation of difference in mean outcome. Rate ratios were used to describe the effect of the intervention on rates. Secondary analyses were conducted post hoc to assess whether the effect of the intervention differed by age group, geographical locality, or timing since last dose of DP. Tests for interaction were conducted, as was a per protocol analysis.

### Ethical approval

The trial was approved by the Ugandan National Council for Science and Technology (UNCST Ref HS 1530), Makerere University School of Biomedical Sciences Research & Ethics Committee (SBS REC 145), London School of Hygiene and Tropical Medicine Ethics Committee (LSHTM Ref 6509), School of Biological and Biomedical Sciences Ethics Committee, Durham University (DU SBBS/EC/STARTv5.0/June15) and University of California, San Francisco Committee on Human Research (UCSF CHR Ref 074826). Sponsorship and insurance were provided by the LSHTM’s Clinical Trials Sub-Committee (Ref QA380). The trial was overseen by an independent Data and Safety Monitoring Board and a Trial Steering Committee.

### Role of the funding source

The funders played no role in the design of the study; in the collection, analysis and interpretation of data; in the writing of the report; or in the decision to submit the paper for publication.

## Results

### IPT intervention recruitment and delivery

Of the 84 primary schools participating in the trial, most were public (85.7%). There were 23,280 children listed on the registers for the 42 intervention schools (mean 554, range 131–1521) vs 21,299 listed for the control schools (mean 507, range 113–1251). As previously reported [24], a total of 89,823 single doses of DP were administered to 10,079 (43.3%) children from 30 June to 12 December 2014; of these, 9286 (92.1%) received at least one full 3-day course of DP. The trial was completed as planned.

### School survey recruitment

**Fig. 2:**
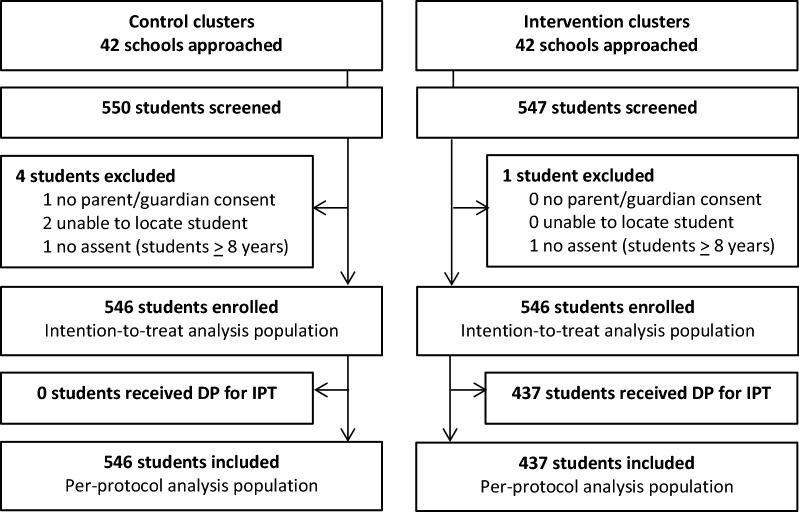
Trial profile

The final school survey was conducted from 13 November to 5 December 2014 (Fig. 2). Characteristics of participants (n = 1092, 13 per cluster) were similar across both study arms (Table 1). A majority of children in both arms resided in rural areas within Jinja district. Most (80%) children enrolled from the intervention arm reported that they were enrolled in the IPT intervention. However, only 378 (69.2%) received any DP, and 356 (65.2%) received at least one full 3-day course of DP, while only 15 (2.8%) received the maximum number of doses in all 6 rounds, before the school survey. Of those who received any DP, there was wide variation in the timing of the last dose of DP before enrollment into the school survey (ranging from 1 to 113 days, with a mean of 15.3 [SD 16] days). Overall, 155 (28.4%) children received DP within 14 days of the survey. Only 38.4% of children reported that they had slept under a bed net the previous night.

**Table 1:** Characteristics of participants surveyed in the final school survey

Characteristic	Control (n = 546)	Intervention (n = 546)
Age, years (mean, SE)	10.5 (0.08)	10.4 (0.08)
5–10 years	276 (50.5%)	290 (53.1%)
11–18 years	270 (49.5%)	256 (46.9%)
Female	285 (52.2%)	260 (47.6%)
Region^a^		
Urban area		
Jinja town	91 (16.7%)	143 (26.2%)
Rural areas		
Buwenge	169 (31.0%)	130 (23.8%)
Kakira/Busedde	117 (21.4%)	130 (23.8%)
Nile River	169 (31.0%)	143 (26.2%)
Enrolled in the intervention^b^	0 (0%)	437 (80.0%)
Slept under a bednet the previous night^b^	194 (35.5%)	225 (41.2%)
Temperature (°C), mean (SE)	37.1 (0.04)	37.1 (0.04)

^a^Comparing urban vs rural areas, adjusting for clustering, *p* value = 0.29

^b^Missing for one child in the intervention arm

### Impact on parasite prevalence

**Fig. 3:**
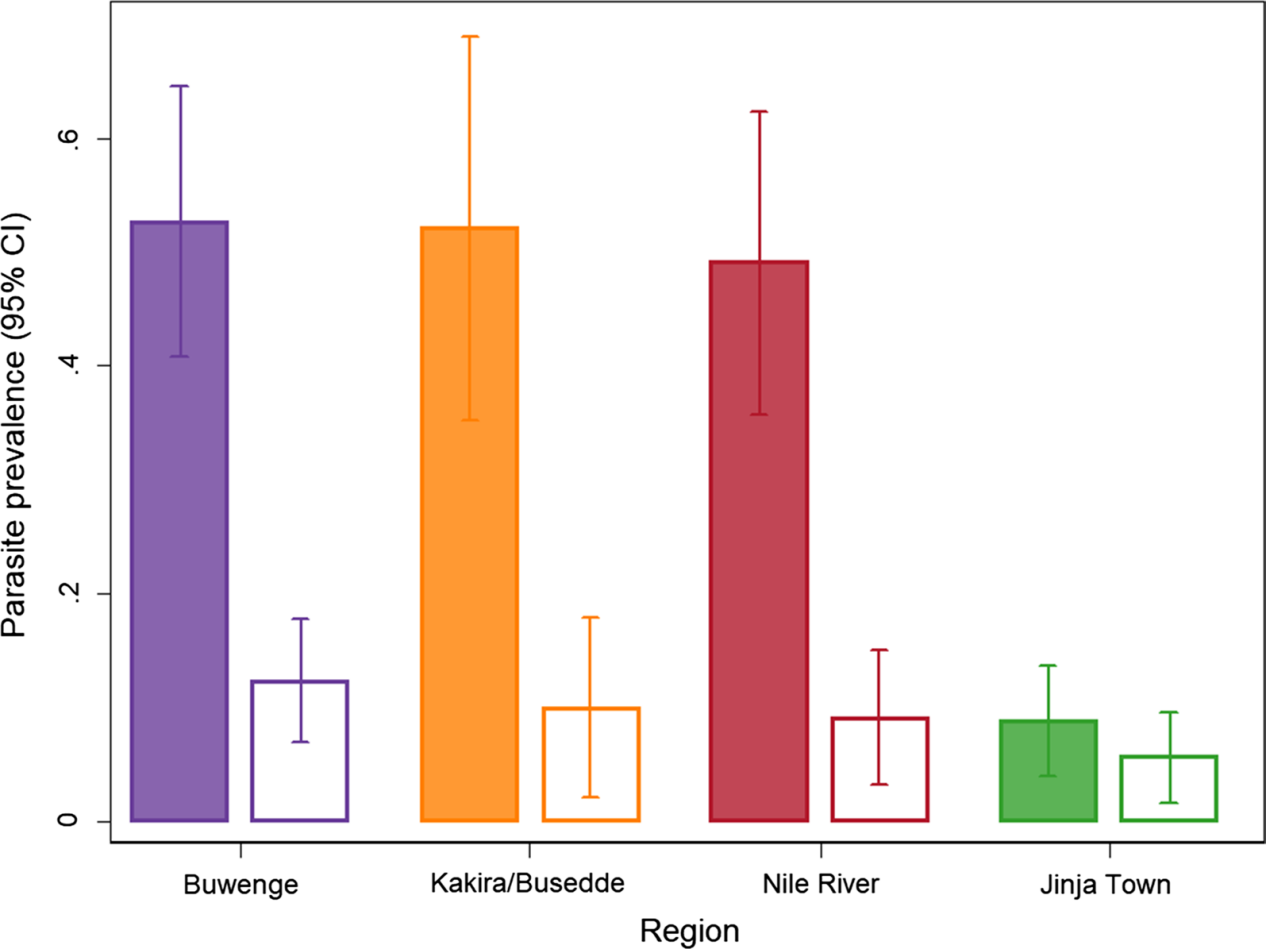
Parasite prevalence by region. For each region, the shaded bars represent the control arm and the open bars represent the intervention arm, both with 95% confidence intervals

The prevalence of parasitaemia by microscopy was lower in the intervention than the control arm (intention-to-treat [ITT]: 9.2% vs 44.1%, adjusted risk ratio [aRR] 0.22 [95% CI 0.16‒0.30], p < 0.001) (Table 2). Similar results were observed when the data were stratified by age, however, differences were noted when stratified by area (Table 2, Fig. 3). In the rural areas, children in the intervention arm were much less likely to be parasitaemic than those in the control arm (ITT: 10.4% vs 51.2%; aRR 0.20 [95% CI 0.14‒0.29, p < 0.001), while in the urban area of Jinja town, the difference between the two study arms was less marked (ITT: 5.6% vs 8.8%; aRR 0.65 [95% CI 0.28‒1.54], p = 0.33). Parasite prevalence varied widely by school (0% to 92%) and the coefficient of variation of parasite prevalence between the control clusters, k, was estimated to be 0.077. In the per-protocol analysis (Table 2), which only included children in the intervention arm if they reported that they had been enrolled in the intervention, similar results were seen, but the difference in parasite prevalence between the intervention and control arms was even more pronounced, with the exception of the results for the urban area.

**Table 2:** Effect of the IPT intervention on parasite prevalence in the final school survey

	n+/N	Prevalence (%)	Unadjusted risk ratio (95% CI)	p	Adjusted risk ratio (95% CI)^a^	p
Intention to treat analysis						
All ages						
Control	241/546	44.1	1		1	
Intervention	50/546	9.2	0.21 (0.14–0.30)	< 0.001	0.22 (0.16–0.30)	< 0.001
5–10 years^b^						
Control	122/276	44.2	1		1	
Intervention	23/290	7.9	0.18 (0.12–0.28)	< 0.001	0.19 (0.13–0.29)	< 0.001
11–18 years^b^						
Control	119/270	44.1	1		1	
Intervention	27/256	10.6	0.24 (0.16–0.36)	< 0.001	0.25 (0.17–0.37)	< 0.001
Urban area^c^						
Control	8/91	8.8	1		1	
Intervention	8/143	5.6	0.64 (0.26–1.58)	0.33	0.65 (0.28–1.54)	0.33
Rural areas^c^						
Control	233/455	51.2	1		1	
Intervention	42/403	10.4	0.20 (0.14–0.30)	< 0.001	0.20 (0.14–0.29)	< 0.001
Per protocol analysis						
All ages						
Control	241/546	44.1	1		1	
Intervention	20/438	4.6	0.10 (0.06–0.17)	< 0.001	0.11 (0.07–0.17)	< 0.001
5–10 years^b^						
Control	122/276	44.2	1		1	
Intervention	9/237	3.8	0.09 (0.04–0.17)	< 0.001	0.09 (0.05–0.19)	< 0.001
11–18 years^b^						
Control	119/270	44.1	1		1	
Intervention	11/201	5.5	0.12 (0.07–0.24)	< 0.001	0.12 (0.06–0.23)	< 0.001
Urban area^c^						
Control	8/91	8.8	1		1	
Intervention	5/106	4.7	0.54 (0.19–1.53)	0.24	0.49 (0.18–1.38)	0.18
Rural areas^c^						
Control	233/455	51.2	1		1	
Intervention	15/332	4.5	0.09 (0.05–0.16)	< 0.001	0.09 (0.05–0.15)	< 0.001

^a^n = 1091, excluded one child with missing bednet information. Adjusted for age group (5–10, 11–18 years); baseline community parasite prevalence (quartiles: 0–13%, 13.01–25%, 25.01–33%, > 33%); sex; bednet use (slept under a bednet the previous night); and region (urban area vs rural areas)

^b^p-value for interaction between trial arm and age group: Intention-to-treat analysis p = 0.18 for the unadjusted model and p = 0.36 for the adjusted model; per-protocol analysis p = 0.41 for the unadjusted model and p = 0.53 for the adjusted model

^c^p-value for interaction between trial arm and region: Intention-to-treat analysis p = 0.02 for unadjusted model and p = 0.09 for adjusted model; per-protocol analysis p = 0.003 for the unadjusted model and p = 0.016 for the adjusted model

To further explore the association between the timing of DP treatment and parasite prevalence, the children in the intervention arm were stratified according to time since the last dose of DP and comparisons were made with the control arm (Table 3). As expected, parasite prevalence was lowest in children who had been treated with DP within the last 28 days, but parasitaemia in children treated more remotely was also reduced. Indeed, even those children from intervention schools who had never received DP had lower parasite prevalence than control children (26.2% vs 44.1%, prevalence ratio 0.57, [95% CI 0.40‒0.81], p = 0.001).

**Table 3:** Association between time since last dose of DP and parasite prevalence

Treatment arm	Days since last dose of DP	Parasite prevalence	PR (95% CI)^a^	p-value
Control	No prior DP	241/546 (44.1%)	Reference group	
Intervention	No prior DP	44/168 (26.2%)	0.57 (0.40–0.81)	0.001
	29–113 days	3/33 (9.1%)	0.20 (0.06–0.64)	0.006
	1–28 days	3/345 (0.9%)	0.02 (0.01–0.10)	< 0.001

^a^Prevalence ratio adjusted for repeated measures within the same cluster

### Impact on secondary outcomes

The prevalence of gametocytaemia was lower in the intervention than the control arm (Table 4). Moreover, children in the intervention arm were less likely to be febrile (elevated temperature or history of fever in the past 48 h) and to have symptomatic malaria (febrile with a positive rapid diagnostic test) than those in the control arm (5.1% vs 35.7%; aRR 0.14 [95% CI 0.08‒0.26], p < 0.001). No differences in prevalence of anaemia or haemoglobin levels were observed between the two groups. School attendance during the study period was similar in both study arms; the total number of days students were absent from intervention schools was 141,383 out of 2,557,776 days vs 116,456 out of 2,106,774 follow-up days in control schools, a rate of 5.5 per 100 days in both groups (rate ratio: 1.02 [95% CI 0.72‒1.43], comparing children in the intervention vs control arm).

**Table 4:** Effect of the IPT intervention on secondary outcomes in the final school survey

	n/N	Prevalence (%)	Crude risk ratio (95% CI)	p	Adjusted risk ratio (95% CI)	p
Prevalence of gametocytaemia						
Control	52/546	9.5	1		1	
Intervention	17/546	3.1	0.33 (0.19–0.56)	< 0.001	0.34 (0.20–0.56)	< 0.001
Prevalence of fever						
Control	307/546	56.2	1		1	
Intervention	110/546	20.2	0.36 (0.25–0.51)	< 0.001	0.35 (0.25–0.50)	< 0.001
Symptomatic malaria						
Control	195/546	35.7	1		1	
Intervention	28/546	5.1	0.14 (0.08–0.26)	< 0.001	0.14 (0.08–0.26)	< 0.001
Prevalence of anaemia						
Control	26/126	20.1	1		1	
Intervention	24/129	18.6	0.90 (0.54–1.51)	0.69	0.82 (0.49–1.38)	0.46
Mean haemoglobin^a^						
Control	126	12.7 (SE 0.14)	0		0	
Intervention	129	12.9 (SE 0.13)	0.15 (− 0.22, 0.52)	0.42	0.19 (− 0.16, 0.54)	0.29

^a^Measured in every 5th participant, results presented as the relative difference in mean values

## Discussion

Intermittent preventive treatment (IPT) is a well-established malaria control intervention that is recommended for certain high-risk populations, but is not current policy for schoolchildren. In this cluster-randomized trial, IPT with DP delivered monthly to primary schoolchildren was associated with a substantial reduction in parasitaemia in all ages, particularly in rural areas. Children in the intervention arm were significantly less likely to have fever and symptomatic malaria, and also less likely to have gametocytaemia. These results contribute to the growing body of evidence that targeting chemoprevention to school-aged children provides important health benefits to individual children. Moreover, by reducing parasitaemia and particularly gametocytaemia, which might otherwise go untreated, IPT of schoolchildren stands to reduce the infectious reservoir of parasites available for onward transmission of malaria. IPT could be integrated into existing school infrastructure and programmes thus expanding the tools available to control malaria in school-aged children and the community as a whole.

In this study, a substantial number of children in the control arm were febrile and were subsequently diagnosed with symptomatic malaria by a positive RDT on the day of the survey; IPT markedly reduced the risk of fever and symptomatic malaria. There is growing awareness of the burden of malaria in school-aged children [10], and the benefits of providing preventive treatment for malaria to children through school-based programmes have been examined in multiple studies [20, 28, 29]. A recent systematic review evaluated the efficacy and safety of IPT of schoolchildren [20]. Five studies, including four individually-randomized trials and one cluster-randomized trial, conducted in 2002–2012 in Kenya, Mali, and Uganda were included, which assessed a variety of anti-malarial regimens. This review suggested that the protective efficacy of IPT against parasitaemia ranged from 49% to 94% depending on the regimen; monthly IPT with DP was most effective (94% protective efficacy [95% CI 93–96%]). Four additional studies of school-based malaria interventions have been published since that review, including one individually-randomized trial of IPT for helminths and malaria with artemether–lumefantrine conducted in Ghana [21], one individually-randomized trial of IPT for malaria with sulfadoxine–pyrimethamine (SP) and SP + piperaquine conducted in the Democratic Republic of Congo [23], one cluster-randomized trial of IPT for malaria with artesunate + SP conducted in Mali [22], and one individually randomized trial of SMC of schoolchildren with artesunate + amodiaquine conducted in Mali [19]. Synthesis of the findings is challenged by heterogeneity of the trial designs, regimens assessed, frequency of treatment, and outcome measures. But, overall, these studies demonstrate that delivering preventive treatment to schoolchildren with combination anti-malarial regimens reduces the risk of parasitaemia, clinical malaria, and anaemia and may improve cognitive indicators.

School-aged children are important contributors to the human infectious reservoir of malaria [13]. In malaria endemic areas, older children, who have acquired some anti-malarial immunity through repeated parasite exposure, may harbour asymptomatic infections which often go untreated [11, 30]. School-aged children often have the highest parasite prevalence within populations [13], and may be more likely to carry gametocytes, the sexual stage of the parasite required for transmission [31, 32]. As a result, older children are a major source of malaria parasites for mosquitoes, perpetuating the transmission cycle [12, 14, 33]. A recent study, which examined the human infectious reservoir for *Plasmodium falciparum* in Burkina Faso and Kenya, found that gametocyte carriage was common in asymptomatic individuals and that children were more likely to infect mosquitoes than adults [14]. After adjusting for mosquito exposure, the proportion of mosquitoes that had been infected by children aged 5–15 years ranged from 41 to 74% in high and moderate transmission areas. In Ethiopia, a study of schoolchildren highlighted the importance of sub-microscopic parasitaemia in this age group, and the positive association between parasite density and gametocyte density for both *P. falciparum* and *Plasmodium vivax* [34]. These findings are supported by another study from Burkina Faso that suggested children are an important component of the infectious reservoir and that sub-microscopic infections contribute to onward transmission [33]. In the main START-IPT trial, a 15% reduction in all-age community-level malaria parasite prevalence was found, despite lower than expected intervention coverage, with the results suggesting reductions as large as 27% or as small as 0% were plausible (19.0% vs 23.1%, adjusted RR 0.85 [95% CI 0.73‒1.00], p = 0.05) [24]. The results of this survey showing that children from the intervention schools had lower parasite prevalence than control children, even if they did not receive DP provide further evidence that IPT with DP may have some community-level effect. Taken together, these findings highlight the important role of school-aged children in malaria transmission and the need to target this age group to control and ultimately eliminate malaria in higher transmission settings.

In this study, IPT with DP was associated with a substantial reduction in parasitaemia in rural areas, but these differences were more modest in the urban area of Jinja town. Parasite prevalence in the control arm varied widely between the rural (51.2%) and urban (8.8%) areas. Reduced transmission and burden of malaria in urban settings is well-described [35], likely due to a reduction in breeding sites, and improved housing construction [36]. Evidence that house design can provide protection from malaria is growing [37, 38]. Several studies have compared houses constructed with modern materials (typically made of brick, concrete, or metal walls, tiled or metal roof, closed eaves) to those constructed with traditional materials (typically made of mud walls, thatched roof, open eaves) [36, 39, 40]. A systematic review of studies from Africa, Asia, and Latin America suggested that residents of ‘modern’ homes are at lower risk of malaria infection and clinical malaria, than residents of ‘traditional’ houses [36]. Given that children residing in urban areas may already be at lower risk of malaria than their rural counterparts, the added benefit of IPT in such settings may be lower. The potential role of IPT of schoolchildren in urban settings may need to be explored further.

Schools provide a potential platform for delivery of health programmes to older children. Schools are already targeted for malaria control interventions in Uganda, acting as sites for targeted delivery of LLINs to schoolchildren. Selected schools located in hard-to-reach areas host facility outreach distribution programmes aiming to maintain universal coverage of LLINs achieved through mass LLIN distribution campaigns led by the Ministry of Health. Schools also serve as a focal point for societal and behaviour change communication. Ugandan schoolchildren have been engaged to deliver malaria intervention messages to their families and friends, thus acting as ‘change agents’ within their communities [41]. Although adding IPT to other school-based programmes would leverage resources and potentially save costs, low uptake of interventions may remain a challenge [42], mass drug administration can feed into existing concerns and mistrust in biomedical interventions [43, 44], and the frequent administration of IPT will need to be taken into consideration [45]. Schools provide an attractive platform for delivery of health programmes, including malaria control interventions, but potential challenges and strategies to address them should be explored and developed through operational research.

In this study, IPT with DP did not have an impact on haemoglobin levels or prevalence of anaemia. The aetiology of childhood anaemia in low- and -middle income countries is multifactorial and complex [46]. However, *P. falciparum* malaria is a well-recognized risk factor for anaemia in malaria-endemic settings [47, 48], along with iron and nutritional deficiencies, parasitic and other chronic infections, and genetic haemoglobin disorders [46]. The reason for the lack of association between IPT with DP and prevalence of anaemia in this study is unclear, however, the limited sampling of only 20% of the study population and the timing of the evaluation (at endline rather than several months after the intervention) may have contributed. Moreover, anaemia predominantly affects children under-five in Uganda and the prevalence of anaemia in this age group, as measured in the Malaria Indicator and Demographic Health Surveys, appears to be declining [3, 49].

This study had several limitations. First, intervention coverage in the study population was suboptimal. Only 65.2% of children enrolled in the survey from the intervention arm received at least one full dose of DP, and only 2.8% received the full 3-day course of DP in all 6 rounds of treatment. However, despite this low coverage, a marked reduction in parasitaemia was achieved in the intervention arm, even in those children who did not receive DP. Of note, IPT coverage in the school surveys, although low, was still higher than in the main trial, which does not rule out potential selection bias. Secondly, in this single cross-sectional survey of schoolchildren, outcomes were only measured at a single timepoint. Prospective evaluation of children in a longitudinally followed cohort might have been preferable, as would longer term follow-up of children to assess sustainability of impact, but resources precluded this type of evaluation. Finally, parasitaemia and gametocytaemia were only measured using microscopy, which may have underestimated prevalence measures. Indeed, there is an increasing appreciation of sub-microscopic infection and more sensitive molecular tests could have been applied, including loop mediated isothermal amplification (LAMP) or polymerase chain reaction (PCR) for asexual parasites [50, 51], and quantitative nucleic acid-based amplification (QT-NASBA) for gametocytes [52].

## Conclusions

In this cluster-randomized trial, IPT with DP delivered to primary schoolchildren reduced parasitaemia, gametocytaemia, fever, and symptomatic malaria. These results contribute to the growing body of evidence that targeting chemoprevention to school-aged children would benefit individual children. Moreover, IPT of schoolchildren stands to reduce malaria transmission by reducing the infectious reservoir of malaria, which could be an important new tool for countries seeking to intensify malaria control on the pathway to elimination. School-based IPT could be integrated with school infrastructure and interventions, thus leveraging existing resources while expanding the toolbox for malaria control. Future research on IPT of schoolchildren should explore strategies to achieve high coverage, approaches to integrate IPT with other school-based interventions, and cost-effectiveness, particularly in urban settings. The potential impact of IPT of schoolchildren on community-level malaria transmission should also be investigated further, ideally at higher coverage levels and in different epidemiological settings.

## Data Availability

The datasets used and/or analysed during the current study are available from the corresponding author on reasonable request.

## Abbreviations

ACT: artemisinin-based combination therapy
aRR: adjusted risk ratio
CI: confidence interval
DP: dihydroartemisin–piperaquine
EIR: entomologic inoculation rate
IPT: intermittent preventive treatment
IRB: institutional review board
IRS: indoor residual spraying
ITT: intention to treat
LAMP: loop mediated isothermal amplification
LLIN: long-lasting insecticide treated bed net
LSHTM: London School of Hygiene and Tropical Medicine
PCR: polymerase chain reaction
PR: prevalence ratio
QT-NASBA: quantitative nucleic acid-based amplification
RDT: rapid diagnostic test
SMC: seasonal malaria chemoprophylaxis
START: School-based Treatment with ACT to Reduce Transmission
SP: sulfadoxine–pyrimethamine
UCSF: University of California, San Francisco
UNCST: Uganda National Council of Science and Technology
WHO: World Health Organization

## References

[1] Bhatt S, Weiss DJ, Cameron E, Bisanzio D, Mappin B, Dalrymple U (2015). The effect of malaria control on *Plasmodium falciparum* in Africa between 2000 and 2015. Nature.

[2] WHO (2018). World Malaria Report 2018.

[3] Uganda Bureau of Statistics (UBOS) and the National Malaria Control Programme of the Ugandan Ministry of Health. Uganda Malaria Indicator Survey 2014–2015. Kampala, Uganda; 2015.

[4] Katureebe A, Zinszer K, Arinaitwe E, Rek J, Kakande E, Charland K (2016). Measures of malaria burden after long-lasting insecticidal net distribution and indoor residual spraying at three sites in Uganda: a prospective observational study. PLoS Med..

[5] Raouf S, Mpimbaza A, Kigozi R, Sserwanga A, Rubahika D, Katamba H (2017). Resurgence of malaria following discontinuation of indoor residual spraying of insecticide in a previously high transmission intensity area of Uganda. Clin Infect Dis.

[6] Okullo AE, Matovu JKB, Ario AR, Opigo J, Wanzira H, Oguttu DW (2017). Malaria incidence among children less than 5 years during and after cessation of indoor residual spraying in Northern Uganda. Malar J..

[7] Rugnao S, Gonahasa S, Maiteki-Sebuguzi C, Opigo J, Yeka A, Katureebe A (2019). LLIN Evaluation in Uganda Project (LLINEUP): factors associated with childhood parasitaemia and anaemia 3 years after a national long-lasting insecticidal net distribution campaign: a cross-sectional survey. Malar J..

[8] WHO. Global Technical Strategy for Malaria 2016–2030. Geneva: World Health Organization; 2015.

[9] WHO, RBM Partnership to End Malaria. High burden to high impact: a targeted malaria response. Geneva: World Health Organization; 2018.

[10] Nankabirwa J, Brooker SJ, Clarke SE, Fernando D, Gitonga CW, Schellenberg D (2014). Malaria in school-age children in Africa: an increasingly important challenge. Trop Med Int Health..

[11] Rodriguez-Barraquer I, Arinaitwe E, Jagannathan P, Kamya MR, Rosenthal PJ, Rek J (2018). Quantification of anti-parasite and anti-disease immunity to malaria as a function of age and exposure. Elife..

[12] Stone W, Goncalves BP, Bousema T, Drakeley C (2015). Assessing the infectious reservoir of falciparum malaria: past and future. Trends Parasitol..

[13] Walldorf JA, Cohee LM, Coalson JE, Bauleni A, Nkanaunena K, Kapito-Tembo A (2015). School-age children are a reservoir of malaria infection in Malawi. PLoS ONE.

[14] Goncalves BP, Kapulu MC, Sawa P, Guelbeogo WM, Tiono AB, Grignard L (2017). Examining the human infectious reservoir for *Plasmodium falciparum* malaria in areas of differing transmission intensity. Nat Commun..

[15] Coalson JE, Cohee LM, Buchwald AG, Nyambalo A, Kubale J, Seydel KB (2018). Simulation models predict that school-age children are responsible for most human-to-mosquito *Plasmodium falciparum* transmission in southern Malawi. Malar J..

[16] Pemberton-Ross P, Smith TA, Hodel EM, Kay K, Penny MA (2015). Age-shifting in malaria incidence as a result of induced immunological deficit: a simulation study. Malar J..

[17] Brooker SJ, Clarke S, Fernando D, Gitonga CW, Nankabirwa J, Schellenberg D, Bundy DAP, Silva N, Horton S, Jamison DT, Patton GC (2017). Malaria in middle childhood and adolescence. Child and adolescent health and development.

[18] Cisse B, Ba EH, Sokhna C, Gomis JF, Dial Y (2016). Effectiveness of seasonal malaria chemoprevention in children under ten years of age in Senegal: a stepped-wedge cluster-randomised trial. PLoS Med..

[19] Thera MA, Kone AK, Tangara B, Diarra E, Niare S, Dembele A (2018). School-aged children based seasonal malaria chemoprevention using artesunate-amodiaquine in Mali. Parasite Epidemiol Control..

[20] Matangila JR, Mitashi P, Inocencio da Luz RA, Lutumba PT, Van Geertruyden JP (2015). Efficacy and safety of intermittent preventive treatment for malaria in schoolchildren: a systematic review. Malar J..

[21] Opoku EC, Olsen A, Browne E, Hodgson A, Awoonor-Williams JK, Yelifari L (2016). Impact of combined intermittent preventive treatment of malaria and helminths on anaemia, sustained attention, and recall in Northern Ghanaian schoolchildren. Glob Health Action..

[22] Clarke S, Rouhani S, Diarra S, Saye R, Bamadio M, Jones R (2017). Impact of a malaria intervention package in schools on Plasmodium infection, anaemia and cognitive function in schoolchildren in Mali: a pragmatic cluster-randomised trial. BMJ Global Health..

[23] Matangila JR, Doua JY, Mitashi P, da Luz RI, Lutumba P, Van Geertruyden JP (2017). Efficacy and safety of intermittent preventive treatment in schoolchildren with sulfadoxine/pyrimethamine (SP) and SP plus piperaquine in Democratic Republic of the Congo: a randomised controlled trial. Int J Antimicrob Agents.

[24] Staedke SG, Maiteki-Sebuguzi C, Rehman AM, Kigozi SP, Gonahasa S, Okiring J (2018). Assessment of community-level effects of intermittent preventive treatment for malaria in schoolchildren in Jinja, Uganda (START-IPT trial): a cluster-randomised trial. Lancet Glob Health..

[25] WHO. Haemoglobin concentrations for the diagnosis of anaemia and assessment of severity. System VaMNI. Geneva: World Health Organization; 2011.

[26] Hayes RJ, Moulton LH (2009). Cluster randomised trials.

[27] Zou G (2004). A modified poisson regression approach to prospective studies with binary data. Am J Epidemiol.

[28] Clarke SE, Jukes MC, Njagi JK, Khasakhala L, Cundill B, Otido J (2008). Effect of intermittent preventive treatment of malaria on health and education in schoolchildren: a cluster-randomised, double-blind, placebo-controlled trial. Lancet.

[29] Nankabirwa JI, Wandera B, Amuge P, Kiwanuka N, Dorsey G, Rosenthal PJ (2014). Impact of intermittent preventive treatment with dihydroartemisinin-piperaquine on malaria in Ugandan schoolchildren: a randomized placebo-controlled trial. Clin Infect Dis.

[30] Nankabirwa J, Wandera B, Kiwanuka N, Staedke SG, Kamya MR, Brooker SJ (2013). Asymptomatic Plasmodium infection and cognition among primary schoolchildren in a high malaria transmission setting in Uganda. Am J Trop Med Hyg.

[31] Zhou Z, Mitchell RM, Kariuki S, Odero C, Otieno P, Otieno K (2016). Assessment of submicroscopic infections and gametocyte carriage of *Plasmodium falciparum* during peak malaria transmission season in a community-based cross-sectional survey in western Kenya, 2012. Malar J..

[32] Coalson JE, Walldorf JA, Cohee LM, Ismail MD, Mathanga D, Cordy RJ (2016). High prevalence of *Plasmodium falciparum* gametocyte infections in school-age children using molecular detection: patterns and predictors of risk from a cross-sectional study in southern Malawi. Malar J..

[33] Ouedraogo AL, Goncalves BP, Gneme A, Wenger EA, Guelbeogo MW, Ouedraogo A (2016). Dynamics of the human infectious reservoir for malaria determined by mosquito feeding assays and ultrasensitive malaria diagnosis in Burkina Faso. J Infect Dis.

[34] Tadesse FG, van den Hoogen L, Lanke K, Schildkraut J, Tetteh K, Aseffa A (2017). The shape of the iceberg: quantification of submicroscopic *Plasmodium falciparum* and *Plasmodium vivax* parasitaemia and gametocytaemia in five low endemic settings in Ethiopia. Malar J..

[35] Robert V, Macintyre K, Keating J, Trape JF, Duchemin JB, Warren M (2003). Malaria transmission in urban sub-Saharan Africa. Am J Trop Med Hyg.

[36] Tusting LS, Ippolito MM, Willey BA, Kleinschmidt I, Dorsey G, Gosling RD (2015). The evidence for improving housing to reduce malaria: a systematic review and meta-analysis. Malar J..

[37] Lindsay SW, Jawara M, Paine K, Pinder M, Walraven GE, Emerson PM (2003). Changes in house design reduce exposure to malaria mosquitoes. Trop Med Int Health..

[38] von Seidlein L, Ikonomidis K, Mshamu S, Nkya TE, Mukaka M, Pell C (2017). Affordable house designs to improve health in rural Africa: a field study from northeastern Tanzania. Lancet Planet Health..

[39] Tusting LS, Bottomley C, Gibson H, Kleinschmidt I, Tatem AJ, Lindsay SW (2017). Housing improvements and malaria risk in sub-Saharan Africa: a multi-country analysis of survey data. PLoS Med..

[40] Rek JC, Alegana V, Arinaitwe E, Cameron E, Kamya MR, Katureebe A (2018). Rapid improvements to rural Ugandan housing and their association with malaria from intense to reduced transmission: a cohort study. Lancet Planet Health..

[41] Uganda Ministry of Health (2014). The Uganda Malaria Reduction Strategic Plan 2014–2020.

[42] Muhumuza S, Olsen A, Nuwaha F, Katahoire A (2015). Understanding low uptake of mass treatment for intestinal schistosomiasis among school children: a qualitative study in Jinja district, Uganda. J Biosoc Sci..

[43] Newby G, Hwang J, Koita K, Chen I, Greenwood B, von Seidlein L (2015). Review of mass drug administration for malaria and its operational challenges. Am J Trop Med Hyg.

[44] Okello G, Jones C, Bonareri M, Ndegwa SN, McHaro C, Kengo J (2013). Challenges for consent and community engagement in the conduct of cluster randomized trial among school children in low income settings: experiences from Kenya. Trials..

[45] Cohee LM, Chilombe M, Ngwira A, Jemu SK, Mathanga DP, Laufer MK (2018). Pilot study of the addition of mass treatment for malaria to existing school-based programs to treat neglected tropical diseases. Am J Trop Med Hyg.

[46] Balarajan Y, Ramakrishnan U, Ozaltin E, Shankar AH, Subramanian SV (2011). Anaemia in low-income and middle-income countries. Lancet.

[47] Korenromp EL, Armstrong-Schellenberg JR, Williams BG, Nahlen BL, Snow RW (2004). Impact of malaria control on childhood anaemia in Africa—a quantitative review. Trop Med Int Health..

[48] Green HK, Sousa-Figueiredo JC, Basanez MG, Betson M, Kabatereine NB, Fenwick A (2011). Anaemia in Ugandan preschool-aged children: the relative contribution of intestinal parasites and malaria. Parasitology.

[49] Uganda Bureau of Statistics (UBOS) and ICF International (2017). Uganda Demographic and Health Survey 2016: key indicators report.

[50] Hopkins H, Gonzalez IJ, Polley SD, Angutoko P, Ategeka J, Asiimwe C (2013). Highly sensitive detection of malaria parasitemia in a malaria-endemic setting: performance of a new loop-mediated isothermal amplification kit in a remote clinic in Uganda. J Infect Dis.

[51] Nankabirwa JI, Yeka A, Arinaitwe E, Kigozi R, Drakeley C, Kamya MR (2015). Estimating malaria parasite prevalence from community surveys in Uganda: a comparison of microscopy, rapid diagnostic tests and polymerase chain reaction. Malar J..

[52] Schneider P, Schoone G, Schallig H, Verhage D, Telgt D, Eling W (2004). Quantification of *Plasmodium falciparum* gametocytes in differential stages of development by quantitative nucleic acid sequence-based amplification. Mol Biochem Parasit..

